# Complement C3 Is the Strongest Predictor of Whole-Body Insulin Sensitivity in Psoriatic Arthritis

**DOI:** 10.1371/journal.pone.0163464

**Published:** 2016-09-22

**Authors:** Francesco Ursini, Salvatore D’Angelo, Emilio Russo, Kassandra Nicolosi, Antonio Gallucci, Agostino Chiaravalloti, Caterina Bruno, Saverio Naty, Giovambattista De Sarro, Ignazio Olivieri, Rosa Daniela Grembiale

**Affiliations:** 1 Department of Health Sciences, University of Catanzaro “Magna Graecia”, Catanzaro, Italy; 2 Rheumatology Department of Lucania, San Carlo Hospital of Potenza and Madonna delle Grazie Hospital of Matera, Potenza, Italy; 3 Pugliese-Ciaccio Hospital, Catanzaro, Italy; 4 Department of Biomedicine and Prevention, University Tor Vergata, Rome, Italy; Katholieke Universiteit Leuven Rega Institute for Medical Research, BELGIUM

## Abstract

**Objectives:**

To evaluate the correlation between inflammatory measures and whole-body insulin sensitivity in psoriatic arthritis (PsA) patients.

**Methods:**

For the present study, 40 nondiabetic PsA patients were recruited. A standard oral glucose tolerance test (OGTT) was performed. The insulin sensitivity index (ISI), insulinogenic index (IGI) and oral disposition index (ODI) were calculated from dynamic values of glucose and insulin obtained during OGTT.

**Results:**

In our study population, mean ISI was 3.5 ± 2.5, median IGI was 1.2 (0.7–1.8), mean ODI 4.5 ± 4.5. In univariate correlation analysis, ISI correlated inversely with systolic blood pressure (sBP) (R = -0.52, p = 0.001), diastolic blood pressure (dBP) (R = -0.45, p = 0.004) and complement C3 (R = -0.43, p = 0.006) and ODI correlated inversely with sBP (R = -0.38, p = 0.02), dBP (R = -0.35, p = 0.03) and complement C3 (R = -0.37, p = 0.02). No significant correlations were found between analyzed variables and IGI. In a stepwise multiple regression, only complement C3 entered in the regression equation and accounted for approximately 50% of the variance of ISI. Using a receiver operating characteristic (ROC) curve we identified the best cut-off for complement C3 of 1.32 g/L that yielded a sensitivity of 56% and a specificity of 96% for classification of insulin resistant patients.

**Conclusions:**

In conclusion, our data suggest that serum complement C3 could represent a useful marker of whole-body insulin sensitivity in PsA patients.

## Introduction

Skin psoriasis (Pso) and psoriatic arthritis (PsA) are inflammatory diseases characterized by an increased risk of major cardiovascular events [1]. Among conventional cardiovascular disease (CVD) risk factors, type 2 diabetes mellitus (T2DM) plays a major role [2]. Glucose homeostasis abnormalities have been considered for a long time a peculiarity of rheumatoid arthritis (RA) [3, 4], but a recent large-scale UK study demonstrated that the risk of incident T2DM in PsA and Pso could be even higher to that conferred by RA [5];moreover a recent work confirmed that abnormal metabolic status is more prevalent in PsA than RA [6].

Metabolic syndrome (MS), a condition characterized by the clustering of different CVD risk factors (including glucose disturbances, visceral adiposity, elevated blood pressure, and abnormal lipid profile), is highly prevalent among patients with Pso [7] and PsA [8]. Although it is now well established that insulin resistance (IR) represents the pathophysiological hallmark of MS [9], the mechanism leading to IR/MS in PsA patients is only partially explained by the abnormal metabolic phenotype [10] and could be accounted for also by the negative impact of TNF-α and other inflammatory cytokines on insulin signaling [11] and altered balance in adipocytokines [12]. The so-called “inflammatory” hypothesis is supported by the evidence that disease-modifying antirheumatic drugs (DMARDs) [13] and biologic agents [14–18] have been demonstrated to improve insulin sensitivity in inflammatory arthritis patients.

The glucose clamp technique by DeFronzo et *al*. [19] is considered the gold standard for the *in-vivo* determination of insulin sensitivity. However, this technique is extremely time consuming and requires experienced operators. For these reason, simple surrogate indexes of insulin sensitivity/resistance calculated from fasting state values such as HOmeostasis Model Assessment of Insulin Resistance (HOMA-IR) [20] or from dynamic testing, such as Matsuda Insulin Sensitivity Index (ISI) [21], are widely used in research applications and clinical practice. However, the evaluation of IR as a novel CVD risk factor in the rheumatologic setting remains still complex to perform. The identification of *clinician-friendly* biomarkers reflecting accurately the metabolic status of the patients could be a simple efficient screening strategy in order to decide which patient should be referred to further evaluation in an appropriate clinical context.

In this view, we previously demonstrated that serum complement C3 is the more accurate inflammatory surrogate of IR in never treated PsA patients [22]. However, our previous study had the limitation of using a surrogate measure of IR, the HOMA-IR, derived from fasting-state only values of glucose and insulin and thus reflecting mainly hepatic IR [23]. The emerging role of skeletal muscle [24] and adipose tissue [25] IR prompted us to perform the present study, in order to evaluate the correlation between inflammation and whole-body insulin sensitivity in PsA patients.

## Materials and Methods

### Patients

The study protocol was approved by the local Ethics Committee (Comitato Etico Azienda Ospedaliera “Mater Domini”, Catanzaro, Italy). For the present study, 40 nondiabetic PsA patients (12 males and 28 females), were recruited at the Rheumatology Outpatient Clinic, Department of Health Sciences, University of Catanzaro “Magna Graecia”, Catanzaro, Italy and at the Rheumatology Department of Lucania, San Carlo Hospital, Potenza, Italy. All patients satisfied the ClASsification criteria for Psoriatic ARthritis (CASPAR) criteria for PsA [26]. Written informed consent was obtained from all patients involved in the present study according to the Declaration of Helsinki. Exclusion criteria were predefined as a past diagnosis of T2DM, polycystic ovary syndrome, infectious or neoplastic diseases; past or current treatment with insulin-sensitizing agents (i.e. metformin or peroxisome proliferator-activated receptor (PPAR) agonists). According to these criteria, 62 consecutive patients were screened and 22 were excluded.

#### Anthropometric measurements

Height and weight were measured with patients wearing light clothing and no shoes, to the nearest 0.1 cm and 0.1 kg respectively. Body mass index (BMI) was calculated with the standard formula:

$$BMI=\frac{Weight}{{Height}^{2}}$$

Waist circumference was assessed with a flexible tape at midpoint between the lowest rib margin and the iliac crest. Blood pressure was measured on the left arm with a mercury sphygmomanometer, with the patient supine and after 5 minutes of rest.

### Disease activity

The Disease Activity Score including 28 joints (DAS28-CRP) was used, evaluating the number of swollen joints (SJC), number of tender joints (TJC), the patients’ global assessment of health measured on a visual analogic scale (GH-VAS, range 0–100 mm), and high sensitivity C-reactive protein plasma concentration (hsCRP, mg/L). A score of DAS28-CRP between 2.6–3.2 indicates low disease activity, > 3.2- ≤ 5.1 moderate and > 5.1 high disease activity. The presence of dactylitis and enthesitis was recorded as a dichotomic variable. Patients were divided in five subsets according to clinical presentation [27]: “polyarthritis” if ≥ five joints involved; “oligoarthritis” if < five joints involved; “DIP predominant” if more than 50% of total joint count being distal interphalangeal joint (DIP) joints; “arthritis mutilans” if patients presented a destructive form of arthritis; and “spine predominant PsA” if inflammatory spinal pain, reduced spinal movements, and/or radiographic sacroiliitis.

### Laboratory evaluation

After overnight fasting, blood samples were obtained for laboratory evaluation. Plasma glucose was measured with automated chemistry analyzer (Cobas 6000/Cobas e411, Roche Diagnostics). Plasma concentration of insulin was determined by chemiluminescence test (Centaur, Siemens HealthCare). Erythrocyte sedimentation rate (ESR) was analyzed by capillary photometry (Test 1, Alifax). High-sensitivity CRP (hsCRP) was measured by immunonephelometry (CardioPhase ® hsCRP, Siemens HealthCare). Serum C3 was measured by nephelometry (Siemens Healthcare Diagnostics, Deerfield, USA).

#### Oral glucose tolerance test (OGTT) and insulin sensitivity

A standard oral glucose tolerance test (OGTT) was performed in all patients. The test was performed according to the recommendations of World Health Organization (WHO).

Briefly, after overnight fasting, the patient was invited to drink a solution with 75 g of anhydrous glucose dissolved in 200 mL of water over a time of 5 minutes; blood samples were collected before and after 30, 60, 90, and 120 minutes, and plasma glucose and insulin concentrations were measured.

Insulin sensitivity index (ISI) was calculated with the equation proposed by Matsuda *et al*. [21] which provides a good approximation of measurements of whole-body insulin sensitivity obtained by the glucose clamp technique:

$$ISI\;(Matsuda)=\frac{10000}{\sqrt{G0\times I0\times Gmean\times Imean}}$$

where ISI, insulin sensitivity index; G0, fasting plasma glucose (mg/dL); I0, fasting plasma insulin (mIU/L); Gmean, mean plasma glucose during OGTT (mg/dL); Imean, mean plasma insulin during OGTT (mIU/L).

Although there are no universally accepted cut-off values for the definition of insulin resistant individuals according to ISI, patients were classified as insulin resistant if ISI ≤ 2.5 as suggested by the authors of the original work [21], that corresponded to the lowest tertile of ISI distribution in the study population. This criterion was subsequently adopted by other groups [28].

Insulinogenic index (IGI), a measure of early phase insulin secretion, defined as the ratio of the increment of insulin to that of plasma glucose 30 minutes after a glucose load, was calculated with the formula [29]:

$$IGI=\frac{{\Delta Insulin}_{0-30\;min}}{{\Delta Glucose}_{0-30\;min}}$$

Oral Disposition Index (ODI), a measure of β-cell function integrated with insulin sensitivity, was calculated with the formula [30]:

ODI=IGI×ISI

### Statistical Analysis

A sample size of at least 38 patients was calculated to detect a correlation coefficient between insulin sensitivity and disease-related variables of 0.50 (calculated on the basis of a previous study by our group [22] with a type I error rate of 0.05 and a type II error rate of 0.10).

Data are expressed as mean ± standard deviation, median (25th–75th percentile), or number (percentage) as appropriate. Continuous variables that were not normally distributed were *ln*-transformed before analysis. The Pearson’s product-moment correlation coefficient and stepwise multiple linear regression were used to evaluate correlation between variables. A receiver operating characteristic (ROC) curve was built to evaluate the predictivity of significant variables on the likelihood of being classified as insulin resistant.

A *p*-value <0.05 was considered statistically significant. All tests were two-tailed. The Statistics Package for Social Sciences (SPSS for Windows, version 17.0, SPSS Inc., Chicago, IL, USA) was used for all analyses.

## Results

General characteristics of the study population are summarized in Table 1. The mean age of the patients was 50.3 ± 11.5 years while disease duration was 3 (1.25–4.00) years. According to clinical subsets 14/40 patients had polyarthritis, 12/40 oligoarthritis, 3/40 DIP predominant disease, 11/40 spine predominant disease. None of the patients recruited had arthritis mutilans. On average, disease activity was moderate with a mean DAS28-CRP of 4.1 ± 0.9. Fifty percent of patients were obese, with a mean BMI of 30.8 ± 5.9 Kg/m^2^.

**Table 1:** General characteristics of the study population.

	PsA (n = 40)
**Age**, years	50.3 ± 11.5
**Males**, n (%)	12 (30)
**PsA subset** • Polyarthritis, n (%) • Oligoarthritis, n (%) • DIP predominant, n (%) • Spine predominant, n (%) • Arthritis mutilans, n (%)	14 (35)12 (30)3 (7.5)11 (27.5)0 (0)
**Enthesitis**, n (%)	8 (20)
**Dactylitis**, n (%)	9 (22.5)
**DAS28-CRP**	4.1 ± 0.9
**Disease duration**, years	3 (1.25–4.00)
**Body Mass Index (BMI)**, kg/m^2^	30.8 ± 5.9
**sBP**, mmHg	128.4 ± 19.3
**dBP**, mmHg	80.7 ± 13.1
**BMI > 30 kg/m**^**2**^, n (%)	20 (50)
**Treatment** • Methotrexate, n (%) • Sulfasalazine, n (%) • Cyclosporin A, n (%) • Infliximab, n (%) • Adalimumab, n (%) • Etanercept, n (%) • Golimumab, n (%) • Corticosteroids, n (%)	23(32.5)2 (5)1 (2.5)2 (5)4 (10)3 (7.5)1 (2.5)9 (22.5)
**ESR**, mm/h	18.2 ± 14.6
***hs*CRP**,mg/L	5.5 ± 6.0
**C4**, g/L	0.26 ± 0.08

**Legend:** PsA, psoriatic arthritis; DIP, distal interphalangeal joint;DAS28-CRP, disease activity score including 28 joints and C-reactive protein; sBP, systolic blood pressure; dBP, diastolic blood pressure; BMI, body mass index; ESR, erythrocyte sedimentation rate; *hs*CRP, high-sensitivity C-reactive protein

Metabolic characteristics of the study population, including glucose and insulin values during OGTT are summarized in Table 2. According to OGTT results, 24 (52.5%) patients could be classified as normal glucose tolerance (NGT), 8 (20%) as impaired fasting glucose (IFG), 3 (7.5%) as impaired glucose tolerance (IGT), 1 (2.5%) as combined IFG/IGT and 4 (10%) were diagnosed with T2DM.In the whole study population, mean ISI was 3.5 ± 2.5, median IGI was 1.2 (0.7–1.8), mean ODI 4.5 ± 4.5.

**Table 2:** Metabolic characteristics of the study population.

	PsA (n = 40)
**Fasting glucose**, mg/dL	95.1 ± 14.9
** • 30 min glucose**, mg/dL	159.4 ± 40.2
** • 60 min glucose**, mg/dL	156.2 ± 45.1
** • 90 min glucose**, mg/dL	139.1 ± 48.5
** • 120 min glucose**, mg/dL	117.0 (98.7–131.3)
**Fasting insulin**, μUI/mL	11.7 (7.0 18.3)
** • 30 min insulin**, μUI/mL	102.9 ± 75.1
** • 60 min insulin**, μUI/mL	119.0 ± 68.8
** • 90 min insulin**, μUI/mL	115.5 ± 81.2
** • 120 min insulin**, μUI/mL	69.5 (43.0–126.5)
**Insulin Sensitivity Index (ISI) ISI ≤ 2.5**	3.5 ± 2.5 18(45)
**Insulinogenic Index (IGI)**	1.2 (0.7–1.8)
**Oral disposition Index (ODI)**	4.5 ± 4.5
**Normal Glucose Tolerance (NGT)**, n (%)	24 (52.5)
**Impaired Fasting Glucose (IFG)**, n (%)	8 (20)
**Impaired Glucose Tolerance (IGT)**, n (%)	3 (7.5)
**IFG *plus* IGT**, n (%)	1 (2.5)
**Type 2 Diabetes**, n (%)	4 (10)

**Legend:** PsA, psoriatic arthritis.

In univariate Pearson’s product-moment correlation analysis, ISI correlated inversely with sBP (R = -0.52, p = 0.001), dBP (R = -0.45, p = 0.004) and complement C3 (R = -0.43, p = 0.006), but not with age, sex, PsA duration, BMI, DAS28-CRP, ESR, C4 or *hs*CRP; ODI correlated inversely with sBP (R = -0.38, p = 0.02), dBP (R = -0.35, p = 0.03) and complement C3 (R = -0.37, p = 0.02) but not with age, sex, PsA duration, BMI, DAS28-CRP, ESR, C4 or *hs*CRP. No significant correlations were found between analyzed variables and IGI (Table 3).

**Table 3:** Univariate correlation analysis between selected variables and measures of insulin sensitivity and β-cell function in the whole study population.

	ISI		IGI		ODI	
	R	*p*-value	R	*p*-value	R	*p*-value
**Age**	-0.6	0.70	-0.31	0.07	-0.28	0.08
**Sex**	-0.9	0.59	0.03	0.86	-0.07	0.68
**Duration**	-0.14	0.38	-0.03	0.84	-0.12	0.45
**BMI**	-0.14	0.38	-0.11	0.53	-0.13	0.41
**sBP**	-0.52	0.001	0.03	0.87	-0.38	0.02
**dBP**	-0.45	0.004	-0.03	0.85	-0.35	0.03
**DAS28-CRP**	-0.30	0.85	0.07	0.73	-0.06	0.70
**ESR**	-0.19	0.27	0.16	0.39	-0.07	0.67
**C3**	-0.43	0.006	-0.07	0.70	-0.37	0.02
**C4**	-0.04	0.81	-0.21	0.23	-0.20	0.22
***hs*CRP**	-0.17	0.29	0.17	0.33	-0.21	0.18

**Legend:** ISI, insulin sensitivity index; IGI, insulinogenic index; ODI, oral disposition index; BMI, body mass index; sBP, systolic blood pressure; dBP, diastolic blood pressure; DAS28-CRP, disease activity score including 28 joints and C-reactive protein; ESR, erythrocyte sedimentation rate; *hs*CRP, high-sensitivity C-reactive protein.

**Fig 1:**
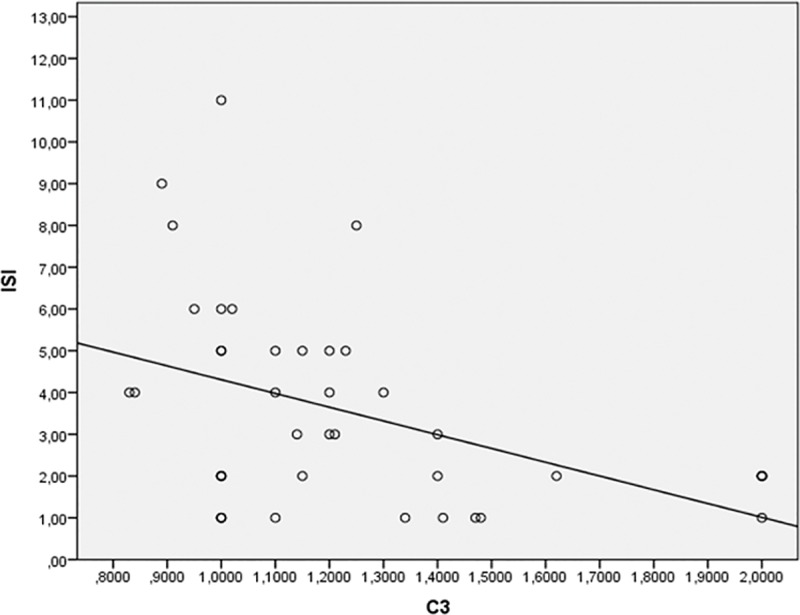
Linear regression of C3 predicting ISI.

Based on univariate correlation analysis above, a stepwise multiple regression model was built using ISI as dependent variable and age, BMI, sBP and complement C3 as predictor variables. At step 1 of the analysis, only complement C3 was entered into the regression equation. Model 1 was statistically significant (F = 5.89, p < 0.02). The standardized β coefficient was -0.50, indicating that approximately 50% of the variance of ISI could be accounted for by C3. A graphical representation of the linear correlation between C3 and ISI is reported in Fig 1.

Finally, we constructed a ROC curve to evaluate the predictivity of complement C3 levels on the likelihood of being classified as insulin resistant according to a literature cut-off of ISI ≤ 2.5 (18/40 or 45% patients). The area under the ROC curve was 0.72 (95% CI: 0.55–0.89, *p* = 0.018). We identified the best cut-off for complement C3 of 1.32 g/L that yielded a sensitivity of 56% and a specificity of 96% for classification of insulin resistant patients.

## Discussion

In this study, we demonstrated that complement C3 is the strongest inflammatory predictor of whole-body insulin sensitivity and adjusted β-cell function in PsA patients.

The main strength of our work, compared to other previously published studies, is that insulin sensitivity was evaluated with the surrogate measure ISI [21], obtained from dynamic values of insulin and glucose measured during OGTT. Other measures of insulin sensitivity/resistance, such as HOMA-IR and the Quantitative Insulin Sensitivity Check Index (QUICKI), are calculated from fasting-state values and mirror mainly hepatic insulin sensitivity; in contrast ISI better reflects whole-body insulin sensitivity, including the contribute from adipose tissue and skeletal muscles [23]. For this reason, ISI is able to identify more accurately clamp-defined subjects with IR when compared with other surrogate measures [31] and performs better than HOMA-IR in predicting the future development of T2DM [32].

Not only IR but also β-cell function, defined as the ability of β-cell to produce sufficient amount of insulin to account for peripheral insulin sensitivity, participates in the pathophysiology of T2DM. In our study, we employed the surrogate measure of β-cell function ODI that has been demonstrated to perform better than fasting-state measures and to predict independently the development of T2DM [30].

Although CRP has been historically considered the more reliable marker of cardiometabolic risk, complement C3 is increasingly emerging as a novel predictor of T2DMand IR [33, 34].

In the general population, C3 correlates with cardiovascular diseases and atherosclerosis [35], especially in heavy smokers [36]. In addition, it correlates with measures of β-cell function [37] and insulin sensitivity and predicts the incidence of future T2DM [38]. A similar correlation between complement C3 and IR has been demonstrated in patients with Pso [39], polycystic ovary syndrome [40] and obesity [41].

Serum C3 is produced mainly by the liver, but other sites, in particular visceral adipose tissue (VAT) [42] contribute significantly to circulating C3 levels. The relationship between visceral fat and C3 is mediated mainly by systemic inflammation and IR [39]. Nevertheless, in our population, no significant association between complement C3 and BMI was observed while waist circumference, the more diffuse measure of visceral adiposity, was not measured. This limitation of our study reduces our ability to speculate on the biological contribute of VAT to complement C3 levels in our population. However, the lack of correlation with BMI is partly explainable with the nature itself of this measure, that represents a global estimate of body mass and do not express accurately visceral adiposity, since some people with normal BMI may have abnormal waist circumference and *vice versa* [43]. In addition, skin cells express complement fractions [44] and C3 has been demonstrated to contribute to skin pathology [45]. Therefore, in the context of Pso and PsA, also skin could contribute to circulating C3 levels.

The increased expression of C3 could be triggered by the systemic inflammatory environment, and several cytokines, such as TNF-α and IL-6, are able to induce C3 expression [46]. Excess of C3 leads to the generation of C3a, that is desarginated by a carboxypeptidase in adipose tissue, generating C3a*des*Arg or acylation-stimulating protein (ASP)[47]. Although previously considered an inactive immune by-product, recent evidence reevaluated its role as a lipogenic hormone. Supporting this hypothesis, circulating levels of ASP are increased in obesity [48] and upon weight loss return to normal values [49]. The binding of ASP to the receptor C5L2 [50] mediates several effects such as increased triacylglycerol synthesis, reduced triglyceride lipolysis, and increased glucose transport [51], thus leading to enhanced fat storage in a vicious circle that leads to incremental IR.

Despite original in methods, our study has some major limitations. First, the relatively low number of patients recruited, albeit appropriately powered to estimate the univariate correlation coefficient, made it impossible to perform more complex multivariate or subgroup analyses to correctly ascertain the influence of other possible confounders. Secondly, the lack of measurements of waist circumference and circulating ASP levels made it impossible to demonstrate the pathophysiological link between circulating C3 levels and insulin sensitivity.

In conclusion, our data suggest that serum C3 > 1.32 g/L could represent a useful marker of insulin sensitivity in PsA patients, easy to use in clinical practice. Larger studies are needed to evaluate the role of C3 in predicting future development of diabetes in PsA and to accurately establish optimal C3 cut-off for the identification of insulin-resistant PsA patients.
